# Ascorbate Peroxidase 2 (APX2) of Chlamydomonas Binds Copper and Modulates the Copper Insertion into Plastocyanin

**DOI:** 10.3390/antiox12111946

**Published:** 2023-10-31

**Authors:** Anna Caccamo, Félix Vega de Luna, Khadija Wahni, Alexander N. Volkov, Jonathan Przybyla-Toscano, Antonello Amelii, Alexandre Kriznik, Nicolas Rouhier, Joris Messens, Claire Remacle

**Affiliations:** 1 Genetics and Physiology of Microalgae, InBios/Phytosystems Research Unit, University of Liège, 4000 Liège, Belgium; anna.caccamo@uliege.be (A.C.); felix_vega@comunidad.unam.mx (F.V.d.L.); jonathan.przybyla-toscano@cea.fr (J.P.-T.); antonello.amelii@univr.it (A.A.); 2 VIB-VUB Center for Structural Biology, 1050 Brussels, Belgium; khadija.wahni@vub.be (K.W.); oleksandr.volkov@vub.be (A.N.V.); 3 Brussels Center for Redox Biology, 1050 Brussels, Belgium; 4 Structural Biology Brussels, Vrije Universiteit Brussel, 1050 Brussels, Belgium; 5 Jean Jeener NMR Centre, Vrije Universiteit Brussel (VUB), 1050 Brussels, Belgium; 6 CNRS, IMoPA and IBSLor, Université de Lorraine, F-54000 Nancy, France; kriznik5@univ-lorraine.fr; 7 INRAE, IAM, Université de Lorraine, F-54000 Nancy, France; nicolas.rouhier@univ-lorraine.fr

**Keywords:** Chlamydomonas, green microalga, ascorbate peroxidase-related, copper-binding motif, peroxidase activity, plastocyanin, structural prediction, ^1^H-NMR

## Abstract

Recent phylogenetic studies have unveiled a novel class of ascorbate peroxidases called “ascorbate peroxidase-related” (APX-R). These enzymes, found in green photosynthetic eukaryotes, lack the amino acids necessary for ascorbate binding. This study focuses on the sole APX-R from *Chlamydomonas reinhardtii* referred to as ascorbate peroxidase 2 (APX2). We used immunoblotting to locate APX2 within the chloroplasts and in silico analysis to identify key structural motifs, such as the twin-arginine transport (TAT) motif for lumen translocation and the metal-binding MxxM motif. We also successfully expressed recombinant APX2 in *Escherichia coli*. Our in vitro results showed that the peroxidase activity of APX2 was detected with guaiacol but not with ascorbate as an electron donor. Furthermore, APX2 can bind both copper and heme, as evidenced by spectroscopic, and fluorescence experiments. These findings suggest a potential interaction between APX2 and plastocyanin, the primary copper-containing enzyme within the thylakoid lumen of the chloroplasts. Predictions from structural models and evidence from ^1^H-NMR experiments suggest a potential interaction between APX2 and plastocyanin, emphasizing the influence of APX2 on the copper-binding abilities of plastocyanin. In summary, our results propose a significant role for APX2 as a regulator in copper transfer to plastocyanin. This study sheds light on the unique properties of APX-R enzymes and their potential contributions to the complex processes of photosynthesis in green algae.

## 1. Introduction

Ascorbate peroxidases (APXs) (EC 1.11.1.11) are heme *b*-containing enzymes belonging to class I peroxidases. They catalyze the reduction of H_2_O_2_ using ascorbate. This reaction involves the formation of the oxidized Compound I (1), which is subsequentially reduced in an intermediate (2) and Compound II (3) by the electron donor ascorbate [1].

(1) $$\mathrm{APX}\;+H_{2}O_{2}\;\to \mathrm{Compound}\;I\;+{\;H}_{2}O\;$$

(2) $$\mathrm{Compound}\;I\;+\mathrm{HS}\;\to \mathrm{Compound}\;\mathrm{II}\;+{\;S}^{.}$$

(3) $$\mathrm{Compound}\;\mathrm{II}\;+\mathrm{HS}\;\to \mathrm{APX}\;+S^{.}+{\;H}_{2}O\;$$

**Figure 1:**
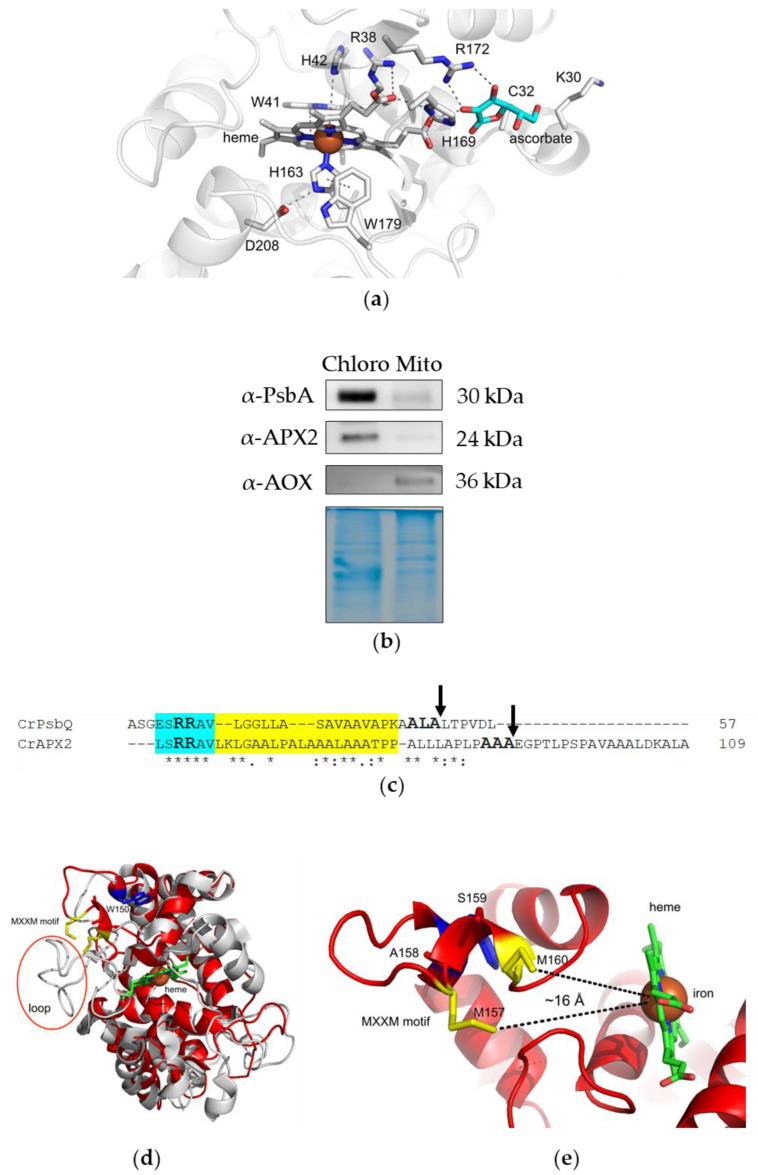
APX2 is localized in the chloroplasts and present specific conserved motifs typical for lumen targeting and metal binding. (**a**) View of the active site of *Pisum sativum* APX1 (PDB: 1APX). Residues associated with the catalytic activity, ascorbate binding, and heme binding are shown. On the distal side, residues R38, W41, and H42 are crucial for catalytic activity, while on the proximal side, H163, D208, and W179 play essential roles. Ascorbate binding is facilitated by R172, with binding stabilization contributed by C32 and K30. Heme binding is associated with residues H163 and H169. The ascorbate placement in *Pisum sativum* APX1 was guided by the sequence alignment with cytosolic ascorbate peroxidase from *Sorghum bicolor* (PDB: 8DJT), resulting in the light-blue representation. The alignment yielded a r.m.s.d. value of 0.283 Å. (**b**) Immunoblots of chloroplast- and mitochondrion-enriched fractions of wt cells (10 μg per lane) developed with antibodies against PsbA, APX2, and alternative oxidase (AOX). Coomassie blue staining of an SDS-PAGE gel was used as loading control. (**c**) Highlight of the TAT motif present in the sequences of APX2 (Cre06.g285150) and PsbQ (Cre08.g372450). Blue indicates the RR sequence, yellow indicates the hydrophobic region, arrows indicate the (putative) cleavage in the lumen. Asterisk (*) indicate fully conserved amino acids, colon (:) indicates conservation between groups of strongly similar properties, period (.) indicates conservation between groups of weakly similar properties. (**d**) Superimposition of the APX2 AF2-model (red) and chloroplast NtAPX (PDB code 1IYN) (gray) shows the absence of the loop (circled in red) facing the heme (green) and the presence of a MxxM motif highlighted in yellow in APX2. The Trp150, which is located close to the MxxM sequence motif, is shown in blue. (**e**) A closer view focused on the region encompassing the copper-binding site of APX2 (MxxM motif) and the heme, revealing an approximate distance of 16 Å. The distance was determined using PyMOL version 2.5.2.

In this reaction, HS represents the substrate, and the S is the oxidized form of the substrate. From the crystal structure of the recombinant pea cytosolic APX, it is observed that the heme is bound to the protein through a bond formed between the iron (Fe) and histidine 163 (H163), as well as a hydrogen bond between the 7-propionate and histidine 169 (H169) (view of the active site in the X-ray structure of Ps_1APX, Figure 1a). The essential active residues within the distal pocket include arginine 38 (R38), tryptophane 41 (W41), and histidine 42 (H42) (Figure 1a). On the proximal side, the catalytic triad consists of histidine 163 (H163), asparagine 208 (D208), and tryptophane 179 (W179) [2] (Figure 1a). Ascorbate binding implicates an interaction close to cysteine 32 (C32) and 6-propionate of the heme [2]. Mutagenesis analyses revealed that arginine 172 (R172) is primarily responsible for ascorbate binding [3], with the additional involvement of lysine 30 (K30) in the binding process [2] (Figure 1a).

Organisms capable of oxygenic photosynthesis, such as plants and algae, have multiple versions of APXs encoded within their nuclear genome, each of which is directed to a distinct organelle [4]. The chloroplast-localized APX isoforms primarily play a role in supporting photosynthetic activity and protection against oxidative damage [5,6]. These enzymes play a crucial role in the water–water cycle. The superoxide generated by the Mehler reaction is subsequently converted into H_2_O_2_ by superoxide dismutase (SOD) located on the stromal side of the thylakoid membranes [7]. During H_2_O_2_ reduction by APXs, ascorbate is oxidized to monodehydroascorbate, which is then reduced back to ascorbate by NADPH-dependent monodehydroascorbate reductases [8,9]. Alternatively, monodehydroascorbate is converted to dehydroascorbate which is then reduced to ascorbate via glutathione-dependent dehydroascorbate reductases [10].

In addition to these well-known APX enzymes, recent sequence and phylogenetic analyses revealed the existence of two new classes, named Ascorbate Peroxidase-Related (APX-R) and Ascorbate Peroxidase-Like (APX-L). Both types of proteins are targeted to the chloroplasts and lack the amino acids responsible for ascorbate binding. However, APX-R contains the heme *b* binding residues, while APX-L does not [11,12]. APX-L (TL-29) of *A. thaliana* has no peroxidase activity and was shown to be associated with photosystem II (PSII) [13,14]. In vitro studies have shown that APX-R (APX6) of *A. thaliana* displays peroxidase activity, but it does not use ascorbate as electron donor [15]. So far, most studies on APX-R have focused on their expression in response to stress conditions [11,16,17,18,19]. In contrast to *A. thaliana,* which possesses eight APXs, including APX6 and TL-29 as cited above, the green alga *Chlamydomonas reinhardtii* (referred to as Chlamydomonas hereafter) has only three APXs: one classical (APX1), one APX-R (APX2), and one APX-L (APX4) [12]. Kuo et al. showed that APX4 is involved in protecting cells from photodamage caused by high light [20]. However, nothing is known about APX2, which is the ortholog of AtAPX6.

Because of the poor characterization of APX-R, we decided to study the APX-R (APX2) from Chlamydomonas. The APX2 sequence exhibits two key features that lead us to propose its potential interaction with plastocyanin: the presence of a metal binding motif (MxxM) [21] and a twin-arginine transport (TAT) motif [22], which is known to direct proteins to the thylakoid lumen. After confirming its localization within chloroplasts and its affinity for binding copper, we propose that APX2 could play a role in regulating the insertion of copper into plastocyanin, the photosynthetic electron carrier which transfers electrons from cytochrome *b_6_f* (*Cyt b_6_f*) to photosystem I (PSI).

## 2. Materials and Methods

### 2.1. Isolation of Chloroplast- and Mitochondrion-Enriched Fractions of Wild-Type Strain

The wt strain (CC-4533 *cw15* mt-) has been ordered from the Chlamydomonas library (https://www.chlamylibrary.org/, accessed on 15 September 2023) [23] and used to isolate chloroplastic and mitochondrial protein fractions. Wt cells grown in mixotrophy (light and acetate) were collected at mid-exponential phase and resuspended in various buffer solutions according to [24,25], respectively. After cell breakage, all the steps were carried out on ice to avoid any degradation and the subcellar fractions were separated and collected after centrifugations on Percoll gradients.

### 2.2. Gel Electrophoresis and Blotting

Mitochondrial and chloroplastic protein fractions were loaded on 12 or 15% Laemmli-SDS-PAGE gel and electroblotted onto PVDF membranes (Cytiva Amersham Hybond, Freiburg, Germany) according to standard protocols. Detection was performed using a Chemiluminescence Western blotting kit (Roche, Mannheim, Germany). The blots were developed using commercially available primary antibodies for PsbA1 protein of PSII (α-D1, 1/10,000, Agrisera AS05 084, Vännäs, Sweden), polyclonal antibodies raised in rabbits against recombinant APX2 (α-APX2, 1/10,000, Proteogenix, Schiltigheim, France), and alternative oxidase (α-AOX,1/27,000, kind gift from Prof S. Merchant). Fluorescence was detected using an iBright FL1000 Imaging System (Invitrogen by Thermo Fisher Scientific, Brussels, Belgium).

### 2.3. Expression of Recombinant APX2 in Escherichia coli and Purification

The sequence encoding the putative mature form of APX2 protein (amino acids 97 to 337) was amplified synthetically (Genecust, Boynes, France). Primers (CrAPX2for 5′-CCCCATATGTCTCCGGCCGTGGCGGCTGCG-3′ and CrAPX2rev CCGGATCCTCACGCCCAGCCCGCCACGCC) were used to clone the sequence into the *Nde*I and *Bam*HI restriction sites of a pET15b vector (Merck, Trosly-Breuil, France) in order to express an N-terminal His-tagged recombinant protein (Figure S1a). The introduction of a His-tag and a cleavage sequence for a protease does not alter the APX-R activity as previously shown [15]. The APX2 protein was expressed in the *Escherichia coli* BL21(DE3) strain co-transformed with the psBET plasmid allowing expression of the AGA and AGG recognizing tRNA. Expression of APX2 was induced at exponential phase with 100 µM of isopropyl 1-thio-β-D-galactopyranoside (IPTG). Of note, the LB culture medium was supplemented with 0.25 mM FeCl_3_ and 1.5 mM 5-aminolevulinic acid 1 h before induction in order to boost heme synthesis as reported [26]. After 4 h induction at 37 °C, bacteria were centrifuged at 6000× *g* for 20 min, and the pellet was resuspended in 30 mM Tris–HCl pH 8.0, 200 mM NaCl lysis buffer containing 10 mM imidazole. Cells were lysed by sonication (three cycles of 1 min, Branson sonifier, 20% amplitude). Insoluble fraction and cellular debris were removed by centrifugation at 40,000× *g* for 25 min. Soluble fraction containing His-tagged APX2 was loaded on a Ni-NTA (Ni^2+^-nitrilotriacetate)–agarose resin equilibrated with 30 mM Tris–HCl pH 8.0, 200 mM NaCl, 10 mM imidazole. After a washing step with 30 mM Tris–HCl pH 8.0, 200 mM NaCl, 10 mM imidazole, His-tagged APX2 was eluted with the same buffer containing 250 mM imidazole. Elution fraction was concentrated using Vivaspin^®^ Turbo columns (10 kDa). Then, a second step of purification by size exclusion chromatography was performed using a HiLoad^®^ 16/600 Superdex^®^ 200 pg column (Cytiva, Marlborough, MA, USA) equilibrated with 30 mM Tris–HCl pH 8.0, 200 mM NaCl. The column was calibrated using the following molecular weight standards: thyroglobulin (670 kDa, 48 mL), β-amylase (200 kDa, 63 mL), bovine serum albumin (66 kDa, 73 mL), and cytochrome c (12.4 kDa, 95 mL). Finally, after a last concentration step, purity and integrity of the purified recombinant protein were analyzed by 15% SDS-PAGE. The concentration of recombinant His-tagged APX2 protein was determined spectrophotometrically using the theoretical molecular extinction coefficient at 280 nm of 24,000 M^−1^ cm^−1^.

### 2.4. Trp Fluorescence Assay

Recombinant His-tagged APX2 was used to perform Trp fluorescence analyses using the SpectraMax^®^ iD3 and iD5 Multi-Mode Microplate Readers. His-tagged APX2 was diluted in 20 mM phosphate buffer, pH 6.0 to 1 μM and titrated with increasing concentrations of CuSO_4_ or of NiCl_2_.6H_2_O dissolved in H_2_O. The change in the Trp fluorescence was monitored at λ_ex_ of 295 nm and following the emission between 335 and 500 nm, with a peak at 350 nm. The dissociation constant (K_D_) for Cu^2+^ binding to APX2 was calculated by fitting the data with the one-site-total-binding equation (GraphPad Prism 9).

### 2.5. Circular Dichroism

Samples for circular dichroism (CD) were prepared at the concentration of 0.2 mg/mL (7.265 μM) for His-tagged APX2 in 20 mM phosphate buffer, pH 6.0 and 50 μM of plastocyanin in 20 mM phosphate buffer, pH 6.0 with 50 mM NaF. The measurements were carried out on a Chirascan Plus spectropolarimeter (Applied Photophysics, Ltd., Leatherhead, UK) or on a MOS-500 spectropolarimeter (BioLogic, Seyssinet-Pariset, France). Data were collected at 1 nm intervals in the wavelength range between 180 nm and 260 nm. A cuvette of 0.01 cm with 30 μL of protein sample for Chirascan Plus spectropolarimeter or 200 μL of protein sample for MOS-500 spectropolarimeter was used for all measurements. Measurements were taken in triplicate, averaging three readings per sample, and each sample spectrum was adjusted for the buffer solution.

### 2.6. APX2/Cu^+^ Stoichiometry Determination

APX2/Cu^+^ stoichiometry was determined as described in [27]. A total of 10 μM of APX2 was incubated with 20 μM of CuSO_4_ in 25 mM MES pH 5.0, 150 mM NaCl in presence of 1 mM DTT to reduce Cu^2+^ into Cu^+^ for 10 min at room temperature with gentle agitation. The samples were concentrated with Amicon Ultra SUB1111 and washed once with 25 mM MES pH 5.0, 150 mM NaCl. The APX2 protein concentration was determined spectrophotometrically using the theoretical molecular extinction coefficient at 280 nm of 24,000 M^−1^ cm^−1^. The copper binding was confirmed by recording a UV-VIS absorption spectrum from 260 nm to 700 nm. The concentration of Cu^+^ was determined by Rapid Gold BCA assay kit (Pierce, Rockford, USA) with a spectrophotometer (Agilent BioTek Synergy Mx Monochromator-Based Multi-Mode Reader with Time-resolved Fluorescence) at 562 nm. Standard curve was prepared using solutions A and B provided by the BCA assay kit. Solution B (copper sulfate, CuSO_4_.5H_2_O) was first prepared in serial dilutions with a factor of two (0, 4.88, 9.77, 19.53, 39.06, 78.13, 156.25, 312.5, 652, 1250, 2500, 5000, 10,000 µM). Each dilution (10 µL) was incubated with the alkaline solution A containing bicinchoninic acid (200 µL) to reduce Cu^2+^ in Cu^+^. The complex formed by Cu^+^ ion chelated with two molecules of bicinchoninic acid was read at 562 nm.

### 2.7. AlphaFold2 Prediction for the Interaction between APX2 and Plastocyanin

The structural prediction was performed with AlphaFold2, powered by ColabFold. The following script was used for running the structural prediction process: colabfold_batch --model-type AlphaFold2-multimer-v2 --num-recycle 48 --amber --use-gpu-relax) [28]. AlphaFold2-multimer-v2 is a specialized version of the AlphaFold2 model tailored for managing protein complexes or multimers. In the prediction process, 48 recycling steps were employed, serving as iterations where the model fine-tuned its predictions to enhance accuracy. The refinement stage utilized AMBER (Assisted Model Building with Energy Refinement), a force field commonly used in molecular dynamics simulations. Additionally, a relaxation process was implemented, optimizing the predicted structures further to attain more realistic and energetically favorable conformations. Sequences were retrieved from Phytozome (https://phytozome-next.jgi.doe.gov/, accessed on 15 September 2023). The copper of plastocyanin and the heme of APX2 were added by superimposition with the 3D structure of a plastocyanin (PDB ID 2PLT) and of the chloroplastic APX from *Nicotiana tabacum* (PDB ID 1IYN), respectively. The approximate distance between the Cu^2+^ in plastocyanin and the center of the heme of APX2 was measured using PyMol version 2.5.2.

### 2.8. Recombinant Plastocyanin

Purified plastocyanin was kindly given by Prof. Michael Hippler and Dr. Yuval Milrad (UMunster) and prepared according to [29]. Apoplastocyanin was obtained by treating holoplastocyanin as described in another study [30] in CHES (N-cyclohexyl-2-aminoethanesulfonic acid) pH 8.5. Then, 5 mM EDTA, 1 M GdnHCl (guanidinium hydrochloride), and 50 µM ascorbate were added, followed by a buffer exchange to 20 mM Na-acetate pH 5.5, 0.2 M NaCl.

### 2.9. NMR Spectroscopy

All proteins except APX2(Cu) were dialyzed against 20 mM MES pH 5, 150 mM NaCl using slide-a-lyzer cassettes of 3.5 kDa (Pierce, Rockford, IL, USA) at 4°C for 2 h. To avoid precipitation of APX2 during the incorporation of copper, we incubated 10 μM APX2 with 20 μM CuSO_4_ in 25 mM MES pH 5.0, 150 mM NaCl, 1 mM DTT, to reduce Cu^2+^ to Cu^+^, by gentle shaking for 10 min. This step was made to be sure that copper would be inserted into APX2, because in these experimental conditions adding directly Cu^2+^ led to APX2 precipitation. This was followed by a buffer exchange to the NMR buffer using 5K cut-off concentrators (vivaspin, Sartorius). To ensure the thorough removal of most DTT, this process was repeated three times. The copper binding was confirmed by recording a UV-VIS absorption spectrum recorded from 260 to 700 nm with a kinetics biophotometer (Eppendorf). The quantification of copper was performed using the Rapid Gold BCA assay kit (Pierce) as described in “APX2/Cu^+^ stoichiometry determination”.

All NMR experiments were performed at 298 K on a Bruker Avance III HD 800 MHz spectrometer equipped with a TCI cryoprobe for enhanced sensitivity. The samples contained 50–100 µM proteins in 20 mM MES pH 5.0, 150 mM NaCl and 6% D_2_O for the lock, and 0.5 mM DSS as the internal standard for chemical shift referencing. The Apx2-bound PC samples were prepared by mixing equal molar concentrations of each protein. A 20 mM buffered stock of CuSO_4_ was used for Cu^2+^ titrations. The 1D ^1^H spectra were acquired with 16 ppm spectral width, 128 or 256 total number of scans, and water suppression using gradient excitation sculpting [31]. NMR experiments were conducted under ambient, aerobic conditions. Consequently, both copper bound APX2 and plastocyanin are in the cupric (Cu^2+^) form. The recording, processing, and analysis of these experiments were carried out using TopSpin 3.6 (Bruker, Rheinstetten, Germany).

### 2.10. Peroxidase Activity Assay

Peroxidase activity of recombinant His-tagged APX2 was measured as oxidation of ascorbate or guaiacol in the presence of increasing H_2_O_2_ concentrations (0, 0.025 mM, 0.1 mM, 0.3 mM, 0.5 mM, 1 mM). Enzyme concentration was used at 0.2 μM with guaiacol at 10 mM, or 1 μM of enzyme with sodium ascorbate at 0.5 mM, in 20 mM potassium phosphate buffer (pH 6.0) [15]. The activity was monitored by recording the changes of absorbance at 470 nm due to formation of tetraguaiacol, or at 290 nm due to consumption of ascorbate [15] in a 1 cm quartz cuvette in a Safas UVmc2-Double Beam spectrophotometer (SAFAS, Monaco, Monaco). The measurements were started with the buffer followed by the addition of guaiacol or ascorbate, of H_2_O_2_, and finally of His-tagged APX2 and followed for two more min. Measurements were repeated twice at each H_2_O_2_ concentration. The rates of H_2_O_2_ consumption were calculated using molar extinction coefficients of 26.6 mM^−1^ cm^−1^ at 470 nm for tetraguaiacol [32] and of 2.8 mM^−1^ cm^−1^ at 290 nm for ascorbate [33]. It was considered that one molecule of formed tetraguaiacol allowed the reduction of two molecules of H_2_O_2_, and one consumed ascorbate allowed the reduction of one molecule of H_2_O_2_ in the reaction. Activity was expressed as equivalents of H_2_O_2_ (μM) consumed per min in the presence of 1 μM of His-tagged APX2.

### 2.11. Structural Predictions

The ColabFold interface [28] was used to construct Multiple Sequence Alignments (MSA) for APX2 (Cre06.g285150) (https://phytozome-next.jgi.doe.gov/, accessed on 15 September 2023) and plastocyanin PCY1 (Cre03.g182551) (https://phytozome-next.jgi.doe.gov/, accessed on 15 September 2023). The following script was used for running the structural prediction process: colabfold_batch --model-type alphafold2_multimer_v3 --num-recycle 48 --amber --use-gpu-relax. Visualization was performed using PyMol version 2.5.2 (https://pymol.org, accessed on 15 September 2023) [34].

## 3. Results

### 3.1. APX2 Resides in Chloroplasts, Presents a TAT Motif for Translocation to the Thylakoid Lumen and a MxxM Motif for Metal Binding

The *APX2* gene model (Cre06.g285150) is 3568 bp-long, composed of seven exons and six introns, and codes for a protein of 337 amino acids. According to the Target-P prediction tool [35], APX2 would be targeted to the chloroplasts. We confirmed this localization by immunoblots on chloroplast- and mitochondrion-enriched fractions (Figure 1b). The purity of the fractions was assessed with markers typical for chloroplasts (PsbA, a subunit of photosystem II) or for mitochondria (alternative oxidase, AOX), and we showed that APX2 predominantly resides in the chloroplasts. The weak signal observed in the mitochondrial fraction was interpreted as a chloroplast contamination, since PsbA was also weakly detected in this fraction (Figure 1b). Moreover, Target-P also predicted the existence of a twin-arginine transport (TAT) motif [22] (Figure 1c), which is known to direct proteins to the thylakoid lumen. It consists of a pair of arginine residues (RR) in the N-terminal region, followed by a hydrophobic region and an AxA cleavage sequence at position 90 (Figure 1c), which would generate a mature protein of 209 amino acids (24 kDa), in accordance with the observed molecular weight on the immunoblot. The TAT-signal sequence present in APX2 is similar to the one present in the oxygen evolving protein PsbQ (Cre08.g372450), an experimentally confirmed luminal protein from Chlamydomonas [36].

Next, Chlamydomonas APX2 was compared with other APX-R and APX from land plants and algae (Figure S1b). The amino acid sequence alignment showed that the known catalytic residues, arginine (R38) and histidine (H42), present in Ps_1APX used as a reference, are conserved. The histidine residue at position 163 (H163), which is essential for heme binding, is also conserved in APX-R sequences. However, the arginine residue important for ascorbate binding (R172) is not present in APX-R, supporting previous results indicating that this class of APX does not use ascorbate as electron donor for H_2_O_2_ reduction [15]. Two specific conserved regions in APX-R sequences were identified. The TAT-signal sequence motif [RR(X…X)A(X)A] mentioned above is found in orthologs from green algae (*Micromonas pusilla* R_Micp, *Chlorella sorokiniana* R_Cs, *Chlamydomonas reinhardtii* R_Cr, *Volvox carteri* R_Vc, *Chlorokybus atmophyticus* R_Ca in Figure S1c) and from some land plants (*Azolla filiculoides* R_Af, *Salvinia cucullata* R_Sc, *Marchantia polymorpha* R_Marp, *Physcomitrium patens* R_Pp, *Selaginella moellendorffii* R_Sm, *Oryza sativa* R_Os, *Zea mays* R_Zm in Figure S1b). A MxxM or MxxH motif (in the case of *Chlorella sorokiniana* R_Cs and *Micromonas pusilla* R_Micp), typical for metal-binding proteins, like Cu^+^, is present in all orthologs from green algae and land plants [21] except for *Marchantia polymorpha* R_Marp and *Salvinia cucullata* R_Sc, both of which exhibit a xxxM motif (Figure S1b). Previously, it has been shown that several MxxM motifs are required for copper binding in copper transporters and copper resistance proteins of *E. coli* [37]. However, in another study conducted on yeast and human transporters [21], the authors proved that a single motif is sufficient for copper binding, albeit with reduced affinity. These two motifs are not present in APX-R from two diatoms, *Thalassiosira pseudonana* and *Phaeodactylum tricornutum* (Figure S1b,c). 

Next, we employed AlphaFold2 [38] to predict the structure of APX2. The average predicted local distance difference test (pLDDT) score of the APX2 model was 91.43, which indicates a highly confident residue structure of APX2. The APX2 predicted structure aligned with the X-ray crystal structure of chloroplast APX from *N. tabacum* (PDB ID 1IYN) (Figure 1d) with an r.m.s.d. value of 0.754 Å. While the fundamental structural fold of APX remains intact, there are significant differences to note. One such distinction is the absence of a loop in APX2, which covers the propionyl tails of the heme group in NtAPX (as illustrated in Figure 1d). This loop is a consistent feature in other traditional APX structures, as depicted in Figure S2a. Instead, the conserved MxxM motif is only present in APX2 (depicted in Figure 1d and Figure S2b). We determined an approximate distance of 16 Å between the MxxM motif for copper binding and the heme of the APX2 (Figure 1e).

### 3.2. Recombinant APX2 Does Not Use Ascorbate as Electron Donor and Binds Copper

**Figure 2:**
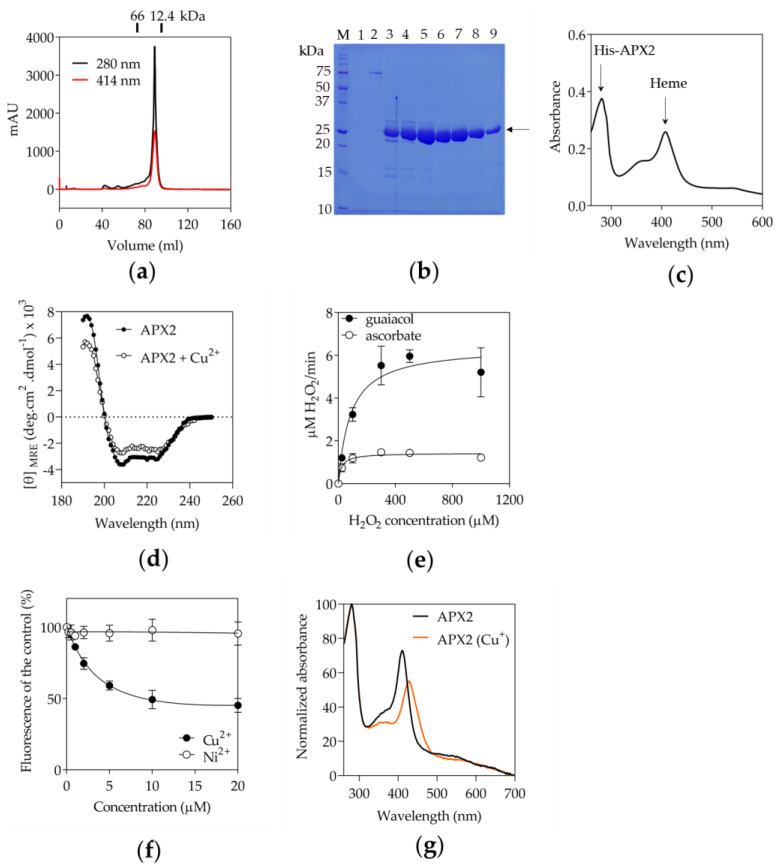
APX2 peroxidase activity relies on guaiacol instead of ascorbate and APX2 binds copper. (**a**) The heme-containing APX2 eluted as a monomer (elution volume at 89 mL) in size exclusion chromatography (SEC, 16/600 Superdex200^®^, Cytiva) based on standard calibration. The molecular masses of proteins used as standards and eluted around APX2 have been indicated (details provided in the method section). Absorbance at 414 nm indicates the presence of a heme. (**b**) Coomassie blue stained SDS-PAGE gel of the fractions eluted from the SEC column of panel. Arrow indicates purified APX2 (**a**). In addition to the molecular weight marker (Precision Plus Protein Unstained Standards, BioRad), lanes 1 and 2 correspond to two fractions that eluted between 40 and 80 mL, whereas lanes 3 to 9 correspond to fractions under the peak. Based on the purity, fractions 6 to 9 have been pooled for subsequent analyses. (**c**) The UV-visible absorption spectrum of APX2 purified protein confirmed the presence of a heme. (**d**) Circular dichroism spectra between 190 and 250 nm of recombinant APX2 (7.3 µM) with and without 300 µM CuSO_4_ in 20 mM phosphate buffer, pH 6.0 are shown. (**e**) Peroxidase activity of recombinant His-APX2 was measured by recording the absorbance of oxidized guaiacol at 470 nm or sodium ascorbate at 290 nm as a function of time. Data were fitted with one site specific binding using GraphPad Prism 9 (*n* = 2 for guaiacol, *n* = 2 for sodium ascorbate). (**f**) Monitoring of the change in tryptophan fluorescence of recombinant His-APX2 with increasing concentrations of Cu^2+^ and Ni^2+^. The changes in fluorescence of tryptophan were monitored at λ_ex_ = 295 nm and emission between 335 and 500 nm, with a peak of maximum fluorescence at 350 nm. The percentage (%) of fluorescence change versus the control at λ_ex_ = 350 nm is shown (*n* = 3). Data were fitted with a reverse hyperbolic equation using GraphPad Prism 9. Values are means with standard deviations. (**g**) Copper binds at the proximity of the heme site. UV-visible absorption spectra of APX2 and copper-loaded APX2 (APX2 (Cu^+^)) are shown.

The observations regarding the lack of the conserved residues for ascorbate binding and the presence of the MxxM sequence motif (Figure S1b) prompted us to express a recombinant His-tagged APX2. The purification process yielded a homogenous form of APX2 (Figure 2a,b), existing as a monomer, as confirmed by gel filtration analysis. The calculated molecular weight (27.5 kDa) is in agreement with the migration distance observed on SDS-PAGE gel (Figure 2b). The UV-visible absorption spectrum showed the presence of the heme (Figure 2a,c). Analysis by circular dichroism (CD) spectroscopy indicated that APX2 exhibited a characteristic folded protein structure (Figure 2d). 

To evaluate the ability of recombinant APX2 to reduce H_2_O_2_, two different electron donors, ascorbate and guaiacol, were tested. The enzyme demonstrated activity in the presence of guaiacol, a well-known electron donor for peroxidases [39] but did not exhibit activity with ascorbate, confirming that APX2 does not utilize ascorbate as an electron donor for the reduction of H_2_O_2_ (Figure 2e and Figure S3). 

Finally, the presence of a MxxM sequence motif led us to investigate the binding of Cu^2+^ to APX2. We decided to check the effect of Cu^2+^ addition on APX2 by evaluating the changes in the secondary structure by CD (Figure 2d) and by measuring the intrinsic tryptophan fluorescence (Figure 2f). While alterations in secondary structure were not definitive, a distinct reduction in tryptophan fluorescence, likely stemming from Trp150 in close proximity to the MxxM motif (Figure 1d), was evident with increasing concentrations of Cu^2+^. We also checked whether the Trp fluorescence change observed after the addition of Cu^2+^ was not due to the binding of Cu^2+^ to the His-tag of APX2, by doing a similar experiment with Ni^2+^. No Trp fluorescence change was observed in the presence of Ni^2+^ (Figure 2f). From these results, a dissociation constant (K_D_) of 3.6 μM was calculated for the binding of Cu^2+^ to APX2. A K_D_-value in the micromolar range hinted in the direction of a copper transfer functionality for APX2. We also determined the copper/APX2 stoichiometry according to [27]. Given that copper enters the lumen in Cu^+^ redox state [40], we aimed to examine the copper/APX2 ratio using Cu^+^. Hence, APX2 was incubated with CuSO_4_ in the presence of the reductant DTT as described in Section 2.6. We found an APX2/Cu^+^ ratio of 1.2 ± 0.1, indicating a one-to-one binding. The copper binding is further evidenced by changes in its UV-vis absorption spectrum (Figure 2g). In particular, the typical Soret band of the heme-containing protein APX2 underwent a shift from 409 nm to 428 nm upon Cu^+^ insertion, indicating alterations in the electronic configuration of the heme. This is likely due to proximity of the heme and Cu-binding sites, as suggested by the predicted structure (Figure 1d,e).

### 3.3. Recombinant APX2 Modulates the Binding of Copper to Plastocyanin

**Figure 3:**
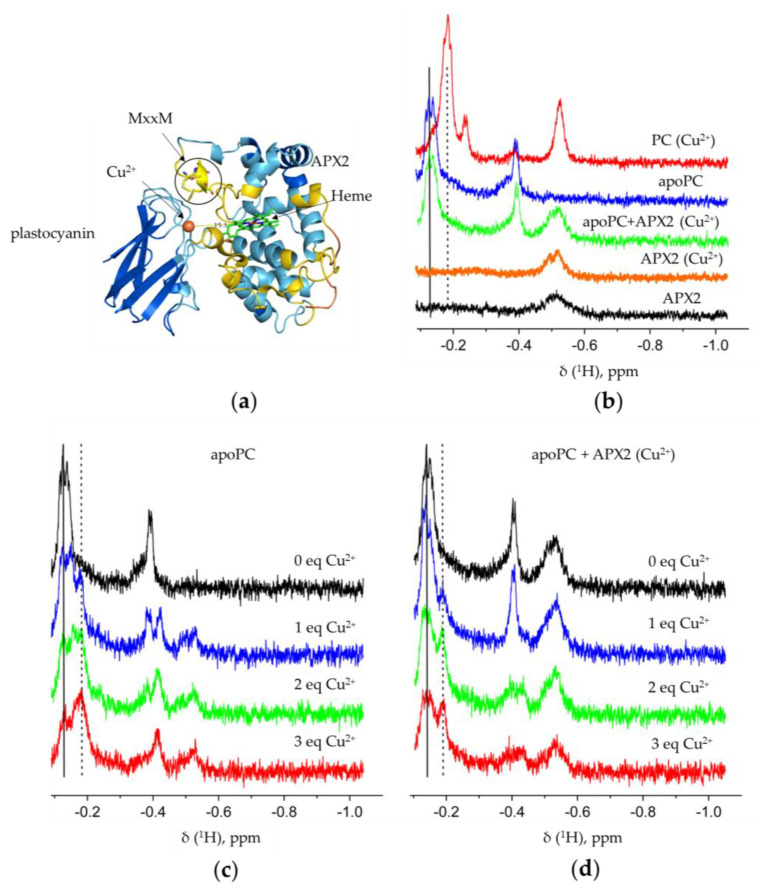
APX2 modulates copper binding in plastocyanin (PC). (**a**) Prediction of the interaction between APX2 and plastocyanin with AlphaFold2. The copper of plastocyanin and the heme of APX2 were added by superimposition of plastocyanin structure (PDB ID 2PLT) and the chloroplastic APX from *Nicotiana tabacum* (PDB ID 1IYN), respectively. The approximate distance (15.1 Å) between the Cu^2+^ in plastocyanin and the center of the heme of APX2 was measured using PyMol2.5.2. (**b**–**d**) Upfield regions of 1D ^1^H NMR spectra of the studied proteins, with the signals of the apo- and holoPC indicated by the vertical solid and dotted lines, respectively. (**c**,**d**) Cu^2+^ titrations of the free (**c**) or APX2-bound (**d**) apoPC.

Considering the ability of APX2 to bind copper and its putative localization in the lumen, we hypothesized that it might interact with plastocyanin, the main copper sink in Chlamydomonas located in the lumen of the chloroplast [41]. This hypothesis was supported by the fact that transcripts of the two genes were expressed at the start of the day phase, when Chlamydomonas cells are subjected to day/night cycles [42] (Figure S4a). This raised the possibility that both proteins could be present at the same time and therefore could interact. Moreover, given the limited knowledge regarding the players needed for copper insertion into plastocyanin [43], we postulated that APX2 might serve as a copper-delivery protein to the apoform of plastocyanin. We utilized AlphaFold2 to predict the interaction between the apoform of plastocyanin and APX2 (as shown in Figure 3a and Figure S4b). To enhance the accuracy of our model, we incorporated copper and heme into the predicted structures through superposition with known structures from the Protein Data Bank, specifically using the plastocyanin structure 2PLT for copper and the APX from *N. tabacum* structure 1IYN for heme (as illustrated in Figure 3a).

To experimentally probe the effect of the APX2-plastocyanin binding on the copper insertion into apoplastocyanin, we have resorted to nuclear magnetic resonance (NMR) spectroscopy (Figure 3b–d). The upfield region of the 1D ^1^H NMR spectrum contains several well-resolved resonances for apo- and holoplastocyanin. The addition of copper-loaded APX2 to apoplastocyanin yielded a spectrum that was the sum of the NMR spectra of the corresponding individual proteins (cf. green, blue, and orange traces in Figure 3b), indicating no direct copper transfer from APX2 to plastocyanin under these NMR conditions. This observation suggested that using purified recombinant proteins may not be the most suitable approach for investigating the mechanism of APX2-mediated copper insertion into plastocyanin. Instead, this process likely requires the specific (redox) conditions present in the cellular environment, which may occur simultaneously with plastocyanin folding or involve other cellular factors. 

Therefore, in the next experiment, we decided to gradually introduce Cu^2+^ ions to apoplastocyanin. This led to an incremental increase in the holoplastocyanin peak and a corresponding decrease in the apoplastocyanin signal (indicated, respectively, by dotted and solid vertical lines in Figure 3c), consistent with the Cu^2+^ binding. Regrettably, we could not achieve saturation in the Cu^2+^ titrations due to protein aggregation occurring at higher Cu^2+^ concentrations. We observed that in the presence of APX2, apoplastocyanin did also bind Cu^2+^, albeit to a lesser degree than in the absence of APX2 (cf. Figure 3c,d). In particular, the intensity ratio between the holo- and apo-plastocyanin peaks consistently remained higher in the free plastocyanin titrations, implying a larger equilibrium fraction of the holoplastocyanin in the absence of APX2. It should be noted that the sub-optimal signal-to-noise ratio of the reported NMR experiments could not be improved due to practical limitations. Indeed, much longer spectral acquisition (which would be required for higher-quality spectra) was precluded by a visible sample aggregation, the extent and onset of which depended on the amount of added Cu^2+^. Thus, the limited sample stability has hampered a more comprehensive study of this system by solution NMR spectroscopy.

Altogether, this analysis suggested that the binding site for Cu^2+^ in plastocyanin might become obstructed due to the formation of a complex between APX2 and plastocyanin. The AlphaFold2 model of the plastocyanin-APX2 interaction (as depicted in Figure 3a) provides insight into the observed inhibitory effect of APX2 on Cu^2+^ insertion into apoplastocyanin. It suggests that APX2 binds in proximity to the Cu-binding site of plastocyanin. 

## 4. Discussion

APX-R enzymes represent a newly identified class of the ascorbate peroxidase family present throughout the eukaryotic green lineage [44]. We showed that APX2 is a plastid enzyme in Chlamydomonas (Figure 1b) and most probably targeted to the luminal side of the thylakoids (Figure 1c and Figure S1b), suggesting that its function might be related to photosynthetic processes. We also demonstrated that APX2 still maintains its peroxidase activity but not dependent on ascorbate as electron donor (Figure 2e), as already shown for the APX6 of Arabidopsis [15]. Further analyses are required to define the substrate in vivo. In addition, APX2 binds copper due to the presence of a MxxM sequence motif (Figure 2d,f,g). 

Copper is one of the most important micronutrients in Chlamydomonas, playing a vital role in photosynthesis. Within the cell, copper exists in two forms: cupric (Cu^2+^) and cuprous (Cu^+^). Copper, initially in the form of Cu^2+^, undergoes reduction by cupric reductase [45], resulting in its insertion into the cell as Cu^+^. The process of copper uptake has been extensively described in Chlamydomonas [46]. Given the toxicity of copper and its potential to generate harmful reactive oxygen species (ROS), various Cu-chaperones are present to mitigate oxidative stress. The chaperone antioxidant 1 (ATX1) has been identified [47]. It is localized in the cytosol and plays a role in distributing copper from copper transporters-like (CTR-like) and/or glutathione (GSH) at the plasma membrane to the secretory pathway. Additionally, there are other putative chaperones, such as the plastid copper chaperone (PCC1), structurally similar to ATX1 and down-regulated in copper deficiency [48]. Another candidate is plastid chaperone 1 (PCH1), derived from an alternative splicing of the copper transporter 2 (CTP2) [27]. In Arabidopsis, PCH1 has been demonstrated to deliver copper to the P_1B_-type ATPase PAA1, ortholog of CTP2, of the inner chloroplast membrane [27]. The transport of copper to the thylakoid lumen is facilitated by CTP4 [41]. Plastocyanin enters the thylakoid lumen unfolded via the SEC translocation system [49] and becomes functional in presence of copper [40]. However, the mechanism of copper insertion into plastocyanin is not yet defined. Based on the fact that APX2 binds copper and is probably targeted to the thylakoid lumen, we hypothesized that APX2 might participate in copper insertion into plastocyanin. While conclusive proof remains elusive, our findings strongly indicate that APX2 binds in the vicinity of the Cu-binding site of plastocyanin, thereby influencing the insertion of copper into this protein (Figure 3b–d). 

Phylogenetic analyses carried out on APXs have revealed that the emergence of APX-R occurred in the basal branches of Chlorophyta, resulting from a process of diversification through the deletion and duplication of the APX enzyme [12]. The scenario varies in other algal phyla. In red algae, for instance, there is no indication of APX-R [12], and no plastocyanin; instead, they utilize the iron-containing carrier cytochrome *c_6_* [50]. Diatoms, such as *P. tricornutum* and *T. pseudonana*, do possess an APX-R, but our research demonstrates that it lacks the distinctive MxxM or MxxH motif required for metal binding and the TAT motif, which hinders its localization to the lumen (Figure S1b,c). Interestingly, in these diatoms, plastocyanin does not serve as the electron carrier between Cyt *b_6_f* and PSI; instead, they employ the iron-containing carrier cytochrome *c_6_* [50]. In some diatoms, such as *Thalassiosira oceanica*, a gene encoding plastocyanin exists, although plastocyanin levels are notably lower compared to those present in green algae, plants, and cyanobacteria [50]. In the case of cyanobacteria, APX activity has been observed, yet no gene encoding for an APX has been identified to date [51]. Land plants also present APX-R as described in [52]. Based on the alignment presented in Figure S1c, the presence of the TAT motif is not definitively evident in some cases, notably in R_Zm (*Zea mays*). This ambiguity is particularly pronounced within the hydrophobic region located between the double arginine (RR) and the putative cleavage site (AxA), raising questions about their potential luminal localization and any potential association with plastocyanin. However, it is worth noting that they do exhibit the MxxM motif, implying a possible conservation of their interaction with copper or plastocyanin. Nevertheless, to substantiate this hypothesis, additional experiments will be necessary.

Examining APX6 of Arabidopsis, a notable observation is the lack of a complete TAT motif, as illustrated in Figure S1c. Instead, it exhibits only the double arginine (RR) without the usual cleavage site (AxA). Nevertheless, the presence of the MxxM motif is evident, as demonstrated in the alignment depicted in Figure S1b. Additionally, structural similarities become evident upon superimposing it with APX2 of Chlamydomonas, as illustrated in Figure S4d. Arabidopsis APX-R (APX6) localizes in the chloroplast [15] and was shown to be translocated from the stroma to vesicles identified as plastoglobuli during photomorphogenesis. These organelles act during senescence and thus participate to APX-R degradation. Other studies on Arabidopsis *apx6* mutant lines provided evidence of the involvement of this enzyme in the senescence process, proposed to be regulated by copper [19]. These findings align with our results, underscoring the connection between copper and APX-R.

## 5. Conclusions

We demonstrated for the first time that the newly classified APX-R enzyme, specifically APX2 of Chlamydomonas, has an affinity for copper binding. This would fit with a role of APX2 in regulating copper insertion into plastocyanin, hinting at potential implications in plastocyanin biogenesis and the integrity of the photosynthetic electron transport chain. Additionally, we have confirmed that this enzyme maintains its peroxidase activity for the reduction of H_2_O_2_ without relying on ascorbate as an electron donor. These results strongly imply that APX-R may have a function distinct from its conventional redox function. To gain a comprehensive understanding of its in vivo function, additional experiments are needed, including the analysis of mutants without APX2 in Chlamydomonas to delve into their physiological traits.

## Data Availability

Not applicable.

## Supplementary Materials

The following supporting information can be downloaded at: https://www.mdpi.com/article/10.3390/antiox12111946/s1, Figure S1: Amino acid sequence alignment of APX and APX-R showing conserved residues in representatives from algae, plants and diatoms; Figure S2: Chlamydomonas APX2 lacks the loop that faces the heme, and APX-R contains the MxxM/H sequence motif; Figure S3: APX2 utilizes guaiacol, but not ascorbate, as electron donor; Figure S4: Further analyses of APX2 and plastocyanin.

## References

[1] Macdonald I.K., Badyal S.K., Ghamsari L., Moody P.C., Raven E.L. (2006). Interaction of ascorbate peroxidase with substrates: A mechanistic and structural analysis. Biochemistry.

[2] Raven E.L. (2003). Understanding functional diversity and substrate specificity in haem peroxidases: What can we learn from ascorbate peroxidase?. Nat. Prod. Rep..

[3] Bursey E.H., Poulos T.L. (2000). Two substrate binding sites in ascorbate peroxidase: The role of arginine 172. Biochemistry.

[4] Caverzan A., Passaia G., Rosa S.B., Ribeiro C.W., Lazzarotto F., Margis-Pinheiro M. (2012). Plant responses to stresses: Role of ascorbate peroxidase in the antioxidant protection. Genet. Mol. Biol..

[5] Shigeoka S., Ishikawa T., Tamoi M., Miyagawa Y., Takeda T., Yabuta Y., Yoshimura K. (2002). Regulation and function of ascorbate peroxidase isoenzymes. J. Exp. Bot..

[6] Foyer C.H., Noctor G. (2011). Ascorbate and glutathione: The heart of the redox hub. Plant Physiol..

[7] Asada K. (1999). THE WATER-WATER CYCLE IN CHLOROPLASTS: Scavenging of Active Oxygens and Dissipation of Excess Photons. Annu. Rev. Plant Physiol. Plant Mol. Biol..

[8] Hossain M.A., Nakano Y., Asada K. (1984). Monodehydroascorbate Reductase in Spinach Chloroplasts and Its Participation in Regeneration of Ascorbate for Scavenging Hydrogen Peroxide. Plant Cell Physiol..

[9] Hossain M.A., Asada K. (1985). Monodehydroascorbate reductase from cucumber is a flavin adenine dinucleotide enzyme. J. Biol. Chem..

[10] Hossain M.A., Asada K. (1984). Purification of Dehydroascorbate Reductase from Spinach and Its Characterization as a Thiol Enzyme. Plant Cell Physiol..

[11] Lazzarotto F., Teixeira F.K., Rosa S.B., Dunand C., Fernandes C.L., de Vasconcelos Fontenele A., Silveira J.A.G., Verli H., Margis R., Margis-Pinheiro M. (2011). Ascorbate peroxidase-related (APx-R) is a new heme-containing protein functionally associated with ascorbate peroxidase but evolutionarily divergent. New Phytol..

[12] Lazzarotto F., Menguer P.K., Del-Bem L.E., Zamocky M., Margis-Pinheiro M. (2021). Ascorbate Peroxidase Neofunctionalization at the Origin of APX-R and APX-L: Evidence from Basal Archaeplastida. Antioxidants.

[13] Granlund I., Storm P., Schubert M., Garcia-Cerdan J.G., Funk C., Schroder W.P. (2009). The TL29 protein is lumen located, associated with PSII and not an ascorbate peroxidase. Plant Cell Physiol..

[14] Lundberg E., Storm P., Schroder W.P., Funk C. (2011). Crystal structure of the TL29 protein from *Arabidopsis thaliana*: An APX homolog without peroxidase activity. J. Struct. Biol..

[15] Lazzarotto F., Wahni K., Piovesana M., Maraschin F., Messens J., Margis-Pinheiro M. (2021). Arabidopsis APx-R Is a Plastidial Ascorbate-Independent Peroxidase Regulated by Photomorphogenesis. Antioxidants.

[16] Tyagi S., Shumayla, Verma P.C., Singh K., Upadhyay S.K. (2020). Molecular characterization of ascorbate peroxidase (APX) and APX-related (APX-R) genes in *Triticum aestivum* L.. Genomics.

[17] Verma D., Upadhyay S.K., Singh K. (2022). Characterization of APX and APX-R gene family in *Brassica juncea* and *B. rapa* for tolerance against abiotic stresses. Plant Cell Rep..

[18] Chen C., Letnik I., Hacham Y., Dobrev P., Ben-Daniel B.H., Vankova R., Amir R., Miller G. (2014). ASCORBATE PEROXIDASE6 protects Arabidopsis desiccating and germinating seeds from stress and mediates cross talk between reactive oxygen species, abscisic acid, and auxin. Plant Physiol..

[19] Chen C., Galon Y., Rahmati Ishka M., Malihi S., Shimanovsky V., Twito S., Rath A., Vatamaniuk O.K., Miller G. (2021). ASCORBATE PEROXIDASE6 delays the onset of age-dependent leaf senescence. Plant Physiol..

[20] Kuo E.Y., Cai M.S., Lee T.M. (2020). Ascorbate peroxidase 4 plays a role in the tolerance of *Chlamydomonas reinhardtii* to photo-oxidative stress. Sci. Rep..

[21] Rubino J.T., Riggs-Gelasco P., Franz K.J. (2010). Methionine motifs of copper transport proteins provide general and flexible thioether-only binding sites for Cu(I) and Ag(I). J. Biol. Inorg. Chem..

[22] Cline K. (2015). Mechanistic Aspects of Folded Protein Transport by the Twin Arginine Translocase (Tat). J. Biol. Chem..

[23] Li X., Patena W., Fauser F., Jinkerson R.E., Saroussi S., Meyer M.T., Ivanova N., Robertson J.M., Yue R., Zhang R. (2019). A genome-wide algal mutant library and functional screen identifies genes required for eukaryotic photosynthesis. Nat. Genet..

[24] Mason C.B., Bricker T.M., Moroney J.V. (2006). A rapid method for chloroplast isolation from the green alga *Chlamydomonas reinhardtii*. Nat. Protoc..

[25] Cardol P., Matagne R.F., Remacle C. (2002). Impact of mutations affecting ND mitochondria-encoded subunits on the activity and assembly of complex I in Chlamydomonas. Implication for the structural organization of the enzyme. J. Mol. Biol..

[26] Sylvestre-Gonon E., Schwartz M., Girardet J.M., Hecker A., Rouhier N. (2020). Is there a role for tau glutathione transferases in tetrapyrrole metabolism and retrograde signalling in plants?. Philos. Trans. R. Soc. Lond. B Biol. Sci..

[27] Blaby-Haas C.E., Padilla-Benavides T., Stube R., Arguello J.M., Merchant S.S. (2014). Evolution of a plant-specific copper chaperone family for chloroplast copper homeostasis. Proc. Natl. Acad. Sci. USA.

[28] Mirdita M., Schutze K., Moriwaki Y., Heo L., Ovchinnikov S., Steinegger M. (2022). ColabFold: Making protein folding accessible to all. Nat. Methods.

[29] Kuhlgert S., Drepper F., Fufezan C., Sommer F., Hippler M. (2012). Residues PsaB Asp612 and PsaB Glu613 of photosystem I confer pH-dependent binding of plastocyanin and cytochrome c(6). Biochemistry.

[30] Ubbink M., Lian L.Y., Modi S., Evans P.A., Bendall D.S. (1996). Analysis of the 1H-NMR chemical shifts of Cu(I)-, Cu(II)- and Cd-substituted pea plastocyanin. Metal-dependent differences in the hydrogen-bond network around the copper site. Eur. J. Biochem..

[31] Hwang T.L., Shaka A.J. (1995). Water Suppression That Works. Excitation Sculpting Using Arbitrary Wave-Forms and Pulsed-Field Gradients. J. Magn. Reson..

[32] Ogola H.J., Kamiike T., Hashimoto N., Ashida H., Ishikawa T., Shibata H., Sawa Y. (2009). Molecular characterization of a novel peroxidase from the cyanobacterium Anabaena sp. strain PCC 7120. Appl. Env. Microbiol..

[33] Kumar P. (2021). Stress amelioration response of glycine betaine and Arbuscular mycorrhizal fungi in sorghum under Cr toxicity. PLoS ONE.

[34] Janson G., Zhang C., Prado M.G., Paiardini A. (2017). PyMod 2.0: Improvements in protein sequence-structure analysis and homology modeling within PyMOL. Bioinformatics.

[35] Almagro Armenteros J.J., Salvatore M., Emanuelsson O., Winther O., von Heijne G., Elofsson A., Nielsen H. (2019). Detecting sequence signals in targeting peptides using deep learning. Life Sci. Alliance.

[36] Mayfield S.P., Schirmer-Rahire M., Frank G., Zuber H., Rochaix J.D. (1989). Analysis of the genes of the OEE1 and OEE3 proteins of the photosystem II complex from Chlamydomonas reinhardtii. Plant Mol. Biol..

[37] Jiang J., Nadas I.A., Kim M.A., Franz K.J. (2005). A Mets motif peptide found in copper transport proteins selectively binds Cu(I) with methionine-only coordination. Inorg. Chem..

[38] Jumper J., Evans R., Pritzel A., Green T., Figurnov M., Ronneberger O., Tunyasuvunakool K., Bates R., Zidek A., Potapenko A. (2021). Highly accurate protein structure prediction with AlphaFold. Nature.

[39] Doerge D.R., Divi R.L., Churchwell M.I. (1997). Identification of the colored guaiacol oxidation product produced by peroxidases. Anal. Biochem..

[40] Aguirre G., Pilon M. (2015). Copper Delivery to Chloroplast Proteins and its Regulation. Front. Plant Sci..

[41] Kropat J., Gallaher S.D., Urzica E.I., Nakamoto S.S., Strenkert D., Tottey S., Mason A.Z., Merchant S.S. (2015). Copper economy in Chlamydomonas: Prioritized allocation and reallocation of copper to respiration vs. photosynthesis. Proc. Natl. Acad. Sci. USA.

[42] Strenkert D., Schmollinger S., Gallaher S.D., Salome P.A., Purvine S.O., Nicora C.D., Mettler-Altmann T., Soubeyrand E., Weber A.P.M., Lipton M.S. (2019). Multiomics resolution of molecular events during a day in the life of Chlamydomonas. Proc. Natl. Acad. Sci. USA.

[43] Merchant S.S., Schmollinger S., Strenkert D., Moseley J.L., Blaby-Haas C.E. (2020). From economy to luxury: Copper homeostasis in Chlamydomonas and other algae. Biochim. Biophys. Acta Mol. Cell Res..

[44] Lazzarotto F., Turchetto-Zolet A.C., Margis-Pinheiro M. (2015). Revisiting the Non-Animal Peroxidase Superfamily. Trends Plant Sci..

[45] Georgatsou E., Mavrogiannis L.A., Fragiadakis G.S., Alexandraki D. (1997). The yeast Fre1p/Fre2p cupric reductases facilitate copper uptake and are regulated by the copper-modulated Mac1p activator. J. Biol. Chem..

[46] Hill K.L., Hassett R., Kosman D., Merchant S. (1996). Regulated copper uptake in *Chlamydomonas reinhardtii* in response to copper availability. Plant Physiol..

[47] Pham K.L.J., Schmollinger S., Merchant S.S., Strenkert D. (2022). Chlamydomonas ATX1 is essential for Cu distribution to multiple cupro-enzymes and maintenance of biomass in conditions demanding cupro-enzyme-dependent metabolic pathways. Plant Direct.

[48] Castruita M., Casero D., Karpowicz S.J., Kropat J., Vieler A., Hsieh S.I., Yan W., Cokus S., Loo J.A., Benning C. (2011). Systems biology approach in Chlamydomonas reveals connections between copper nutrition and multiple metabolic steps. Plant Cell.

[49] Smeekens S., Bauerle C., Hageman J., Keegstra K., Weisbeek P. (1986). The role of the transit peptide in the routing of precursors toward different chloroplast compartments. Cell.

[50] Castell C., Rodriguez-Lumbreras L.A., Hervas M., Fernandez-Recio J., Navarro J.A. (2021). New Insights into the Evolution of the Electron Transfer from Cytochrome f to Photosystem I in the Green and Red Branches of Photosynthetic Eukaryotes. Plant Cell Physiol..

[51] Miyake C., Michihata F., Asada K. (1991). Scavenging of Hydrogen Peroxide in Prokaryotic and Eukaryotic Algae: Acquisition of Ascorbate Peroxidase during the Evolution of Cyanobacteria. Plant Cell Physiol..

[52] Dunand C., Mathe C., Lazzarotto F., Margis R., Margis-Pinheiro M. (2011). Ascorbate peroxidase-related (APx-R) is not a duplicable gene. Plant Signal Behav..

