# Supplementary material for: Ascorbate Peroxidase 2 (APX2) of Chlamydomonas Binds Copper and Modulates the Copper Insertion into Plastocyanin
Source: Antioxidants (Basel). 2023 Oct 31;12(11):1946. doi: 10.3390/antiox12111946 (PMC10669542; doi:10.3390/antiox12111946)
Supplement: Supplementary file 1 [file antioxidants-12-01946-s001.zip › antioxidants-2645516-supplementary.pdf]

Supplementary Materials

Recombinant His-tagged APX2

MGSSHHHHHHSSGLVPRGSHMSPAVAAALDKALAKNIPKTKTAVALRLAFHDAATFSAGAKDGGLNA
SIQYELDRPENFGLKRGWRIIEQVRADLKGTAAEGVVTDADLVALAGAFAVRLCGGPAIPLPIGRPVAAA
ARQDPPGRMPSENASAAELKANFAAKGLSVQEMVALSGAHTLGSKGFGDPVTFDNAYYVALLQKPWN
NTKDAMASMIGLPSDHVLPDDPDCLPVIQRYAADQDLFFRDFSAAYIKMCGLGVAGWA

APX2 (Cre06.g285150)

MSTSVLSHRSLSNCTRHGNPRRACRVATHAKLRDLSQWRQEGSGTSEPAVAPELSRRAVLKLGAALPALA
AALAAATPPALLLAPLPAAAEGPTLPSPAVAAALDKALAKNIPKTKTAVALRLAFHDAATFSAGAKDGG
LNASIQYELDRPENFGLKRGWRIIEQVRADLKGTAAEGVVTDADLVALAGAFAVRLCGGPAIPLPIGRPVA
AAARQDPPGRMPSENASAAELKANFAAKGLSVQEMVALSGAHTLGSKGFGDPVTFDNAYYVALLQKP
WNNTKDAMASMIGLPSDHVLPDDPDCLPVIQRYAADQDLFFRDFSAAYIKMCGLGVAGWA

(**a**)

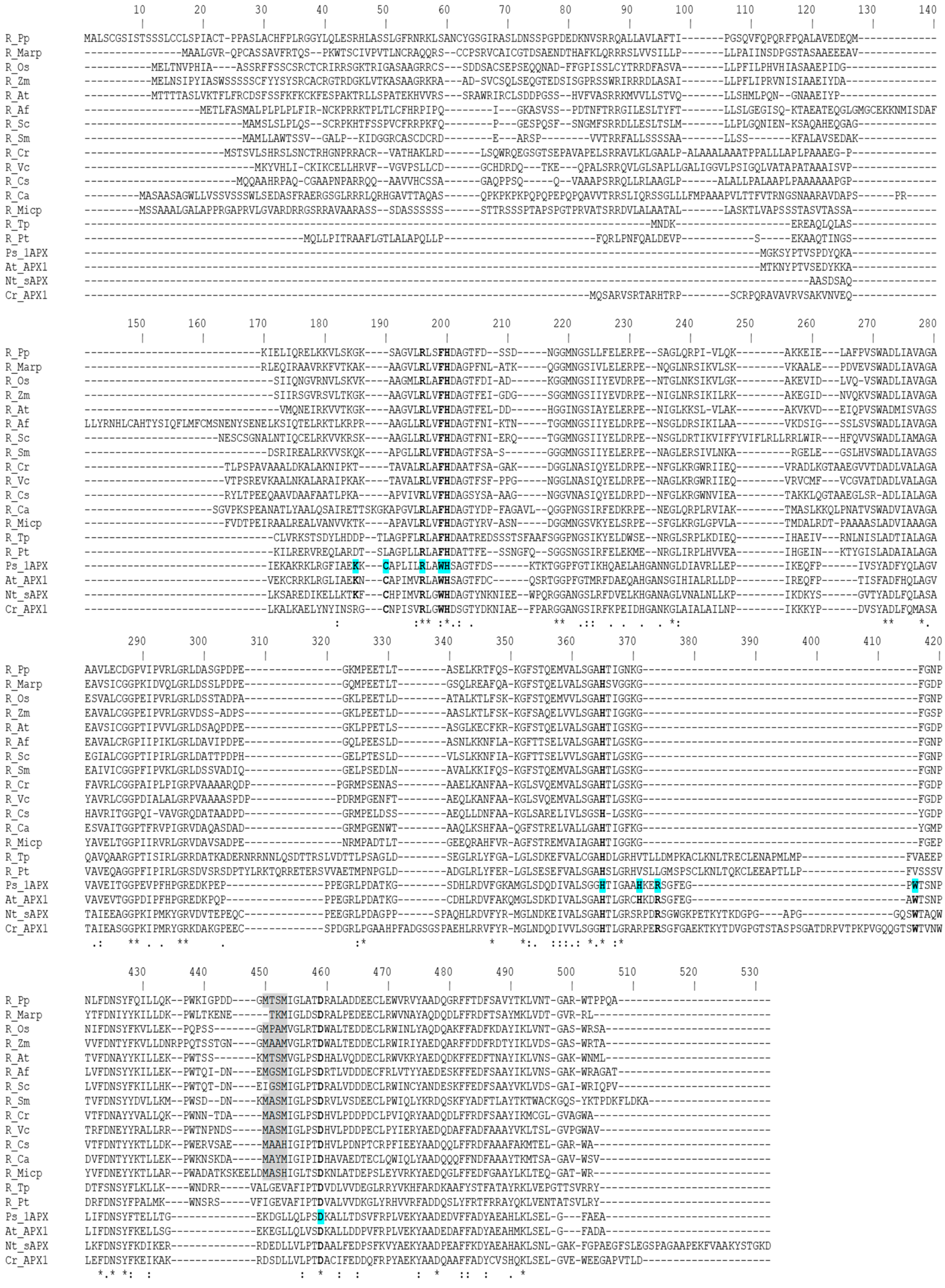

(b)

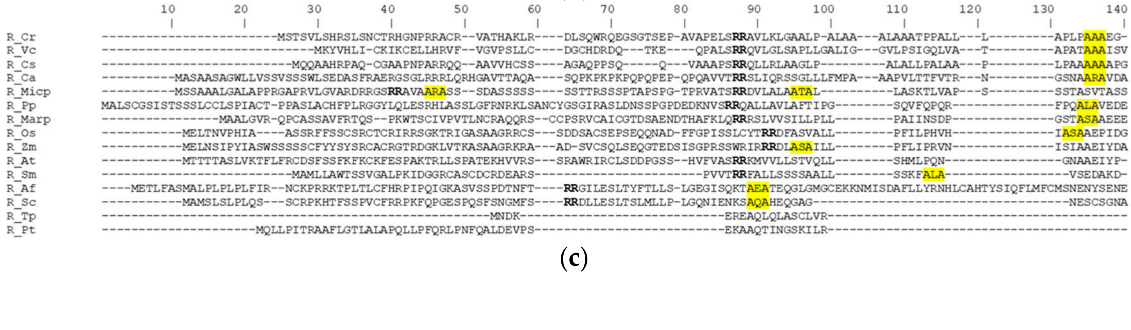

(c)

Figure S1. Amino acid sequence alignment of APX and APX-R showing conserved residues in representatives from algae, plants and diatoms.
(**a**) Sequence of recombinant His-tagged APX2 expressed in *E. coli*. The His-tag is underlined in blue and the sequence for cleavage by thrombin in violet. The sequence of APX2 (Cre06.g285150) from Phytozome is also shown and the targeting signal that has been removed in recombinant His-tagged APX2 is in gray.

(**b**) The amino acid sequence alignment of APX-R from selected photosynthetic organisms (green algae, diatoms, plants) was performed using ClustalW (https://npsa-pbil.ibcp.fr/cgi-bin/npsa_automat.pl?page=/NPSA/npsa_clustalw.html, accessed on 15 September 2023). The organisms were chosen based on [1] and the sequences retrieved from the RedOxiBase (https://peroxibase.toulouse.inra.fr/, accessed on 15 September 2023). The sequences for classical APXs were retrieved by blastp analysis using AtAPXs. Double arginine for TAT-motif is highlighted in bold (CrAPX2 as RedoxiBase reference: 56RR57, putative cleavage site is highlighted in yellow). Conserved amino-acid residues are highlighted in bold. The ones of the reference sequence from *Pisum sativum* Ps_1APX are in blue and bold, however, due to the alignment the exact position of these residues as described in [2] in the crystal structure has shifted (the essential active residues are **R38 -> R196**, **W41 -> W199, H42 -> H200**, **H163 -> H365**, **W179 -> W416, D208 -> D459**; the primary residue responsible for ascorbate binding is **R172 -> R374** together with the contribution of **C32 -> C190** and **K30 -> K185**; **H163 -> H365** and **H169 -> H371** are the heme-binding residues). In grey is highlighted the **MxxM or MxxH** motif typical of metal binding.

RedOxiBase Entry ID are the following: **R_Tp** *Thalassiosira pseudonana* APX-R 2559; **R_Pt** *Phaeodactylum tricornutum* APX-R 8208; **R_Micp** *Micromonas pusilla* APX-R 8359; **R_Cs** *Chlorella sorokiniana* APX-R 15183; **R_Cr** *Chlamydomonas reinhardtii* APX-R 7433; **R_Vc** *Volvox carteri* APX-R 8357; **R_Ca** *Chlorokybus atmophyticus* APX-R 7707; **R_Af** *Azolla filiculoides* APX-R 16514; **R_Sc** *Salvinia cucullate* APX-R 16550; **R_Marp** *Marchantia polymorpha* APX-R 5764; **R_Pp** *Physcomitrella patens* APX-R 5761; **R_Sm** *Selaginella moellendorffii* APX-R 7143; **R_At** *Arabidopsis thaliana* APX-R 3952; **R_Os** *Oryza sativa* APX-R 3961; **R_Zm** *Zea mays* APX-R 5183.

(**c**) ClustalW (https://npsa-pbil.ibcp.fr/cgi-bin/npsa_automat.pl?page=/NPSA/npsa_clustalw.html, accessed on 15 September 2023) was used to perform the alignment of the APX-R from selected organisms (green algae, diatoms, plants). Sequences of APX-R were retrieved on RedOxiBase (https://peroxibase.toulouse.inra.fr/). Entry ID are the following: **R_Tp** *Thalassiosira pseudonana* APX-R 2559; **R_Pt** *Phaeodactylum tricornutum* APX-R 8208; **R_Micp** *Micromonas pusilla* APX-R 8359; **R_Cs** *Chlorella sorokiniana* APX-R 15183; **R_Cr** *Chlamydomonas reinhardtii* APX-R 7433; **R_Vc** *Volvox carteri* APX-R 8357; **R_Ca** *Chlorokybus atmophyticus* APX-R 7707; **R_Af** *Azolla filiculoides* APX-R 16514; **R_Sc** *Salvinia cucullata* APX-R 16550; **R_Marp** *Marchantia polymorpha* APX-R 5764; **R_Pp** *Physcomitrella patens* APX-R 5761; **R_Sm** *Selaginella moellendorffii* APX-R 7143; **R_At** *Arabidopsis thaliana* APX-R 3952; **R_Os** *Oryza sativa* APX-R3961; **R_Zm** *Zea mays* APX-R 5183. Putative twin-arginine-translocation motif (TAT) is highlighted: double arginine (RR) for chloroplast translocation is highlighted in bold and the AxA cleavage site for luminal translocation is highlighted in yellow (as checked using TargetP-2.0, https://services.healthtech.dtu.dk/services/TargetP-2.0/, accessed on 30 October 2023). The AxA cleavage site is not clear for R_Micp, R_Zm, R_Af and R_Pp, and is lacking for R_At. Differences in the length of the hydrophobic region between the RR and the AxA cleavage site can be noticed between algal and land plant sequences. The members in the two diatoms **R_Tp** *Thalassiosira pseudonana* and **R_Pt** *Phaeodactylum tricornutum* do not present the TAT motif.

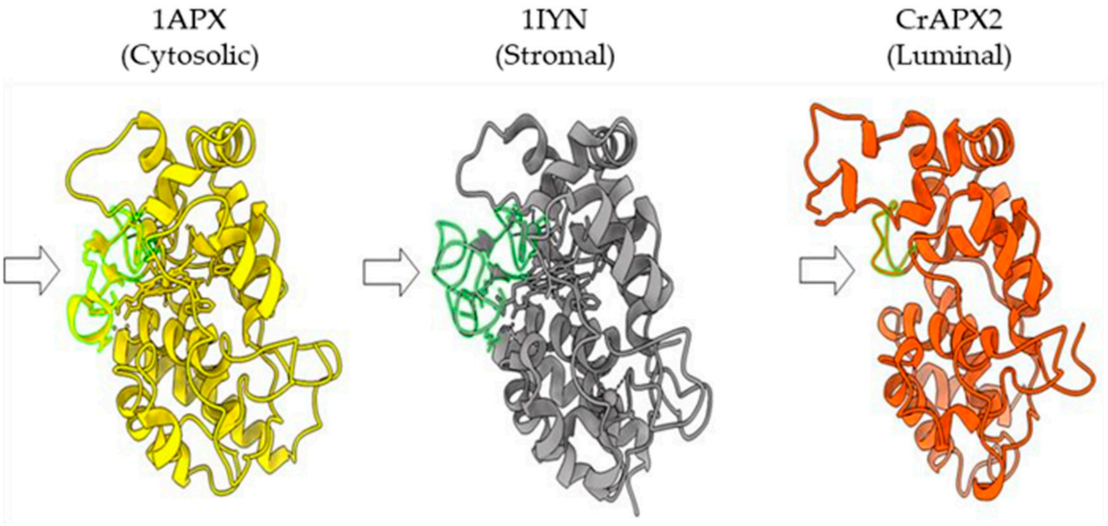

(**a**)

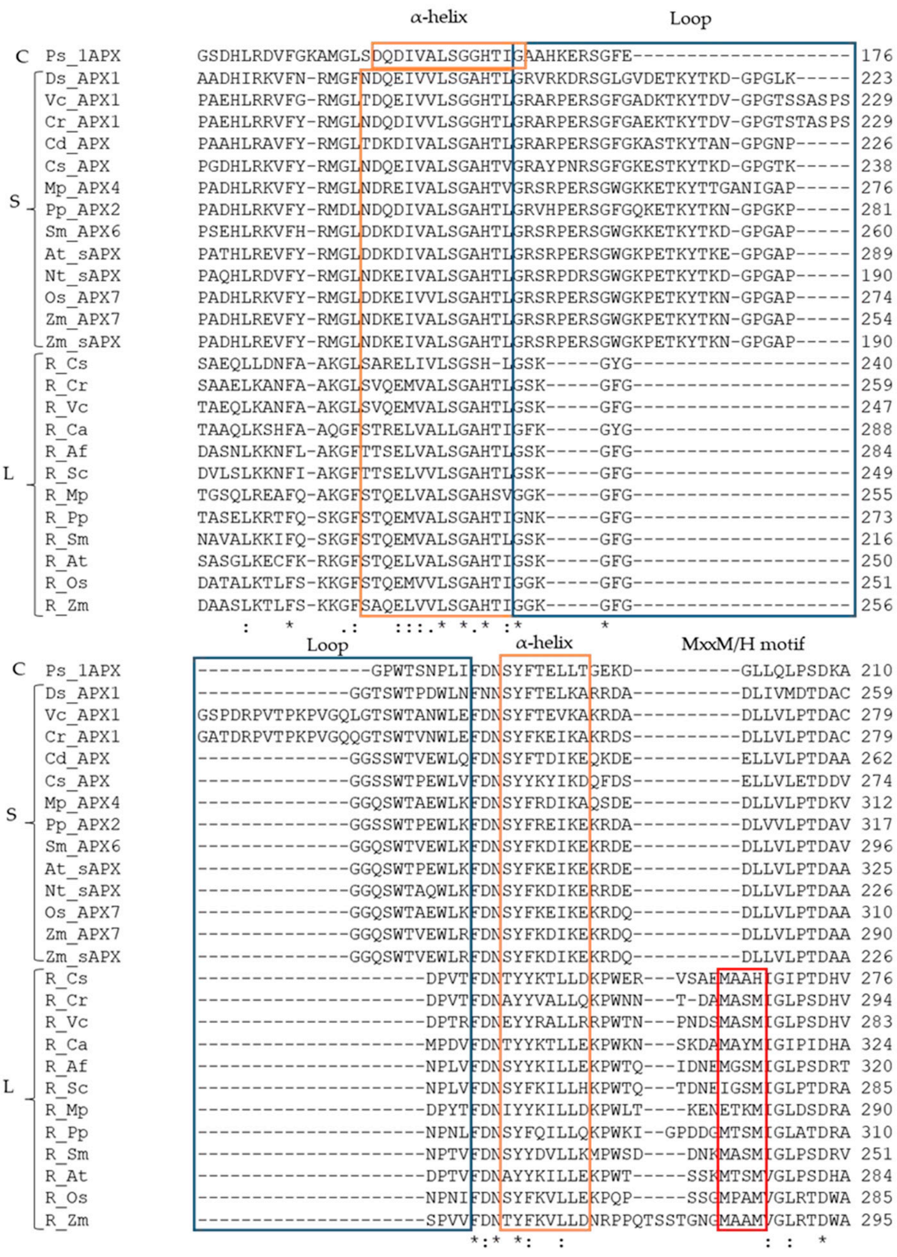

(**b**)

Figure S2. Chlamydomonas APX2 lacks the loop that faces the heme, and APX-R contains the MxxM/H sequence motif. (**a**) Structural comparison of classical cytosolic and chloroplastic APXs (respective PDB ID 1APX for *Pisum sativum* APX1; 1IYN for *Nicotiana tabacum* sAPX) with the predicted structure of Chlamydomonas APX2 [3]. The loop covering the heme propionyl tails present in the cytosolic and stromal APX enzymes is absent in CrAPX2, in which there is a solvent-exposed MxxM or MxxH motif. (**b**) Partial amino acid sequence alignment showing differences in the secondary structures (including the loop facing the heme or the MxxM or MxxH motif) between cytosolic (C), stromal (S) and luminal (L) isoforms (APX-R). Sequences of cytosolic and stromal isoforms were retrieved by a blastp search using stromal APX from *A. thaliana* (AtsAPX). The sequences of chloroplast APX isoforms used are the same as in Figure S1 and they are compared with the cytosolic isoform APX1 of *P. sativum*, from which the helices and loops are defined by crystal structure.

RedOxiBase Entry ID are the following: **R_Cs** *Chlorella sorokiniana* APX-R 15183; **R_Cr** *Chlamydomonas reinhardtii* APX-R 7433; **R_Vc** *Volvox carteri* APX-R 8357; **R_Ca** *Chlorokybus atmophyticus* APX-R7707; **R_Af** *Azolla filiculoides* APX-R 16514; **R_Sc** *Salvinia cucullata* APX-R 16550; **R_Mp** *Marchantia polymorpha* APX-R 5764; **R_Pp** *Physcomitrella patens* APX-R 5761; **R_Sm** *Selaginella moellendorffii* APX-R 7143; **R_At** *Arabidopsis thaliana* APX-R 3952; **R_Os** *Oryza sativa* APX-R 3961; **R_Zm** *Zea mays* APX-R 5183.

GenBank/NCBI accession numbers are as follows: **Ps_1APX** *Pisum sativum* 1APX XP_050898393.1; **Ds_APX1** *Dunaliella salina* APX1 QBY35580; Vc_APX1 Volvox carteri APX1 XP_002953608.1; **Cr_APX1** *Chlamydomonas reinhardtii* APX1 XP_001701947.1; **Cd_APX** *Chlorella desiccata* APXhypot KAG7671850.1; **Cs_APX** *Coccomyxa subellipsoidea* APX XP_005643275.1; **Mp_APX4** *Marchantia polymorpha* APX4 PTQ42354.1; **Pp_APX2** *Physcomitrella patens* APX2 XP_024381690.1; **Sm_APX6** *Selaginella moellendorffii* APX6 XP_002986232.1; **At_sAPX** *Arabidopsis thaliana* sAPX NP_001319883.1; **Nt_sAPX** *Nicotiana tabacum sAPX* 1IYN_A; **Os_APX7** *Oryza sativa* APX7 NP_001389298.1; **Zm_APX7** *Zea mays* APX7 NP_001132683.1; **Zm_sAPX** *Zea mays* sAPX ONM17334.1.

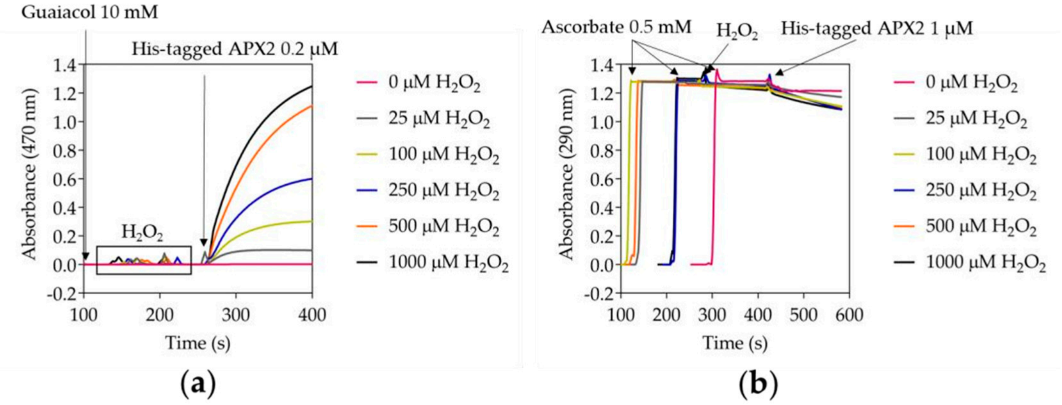

Figure S3. APX2 utilizes guaiacol, but not ascorbate, as electron donor. Peroxidase activity progress curves are shown. (**a**) Oxidation of guaiacol monitored at 470 nm as a function of time in the presence of recombinant APX2 and increasing H_2O_2 concentrations and (**b**) Oxidation of sodium ascorbate followed at 290 nm as a function of time in the presence of recombinant APX2 and increasing H_2O_2 concentrations. Arrows pointing at the position where guaiacol, ascorbate, H_2O_2, and His-tagged APX2 were added.

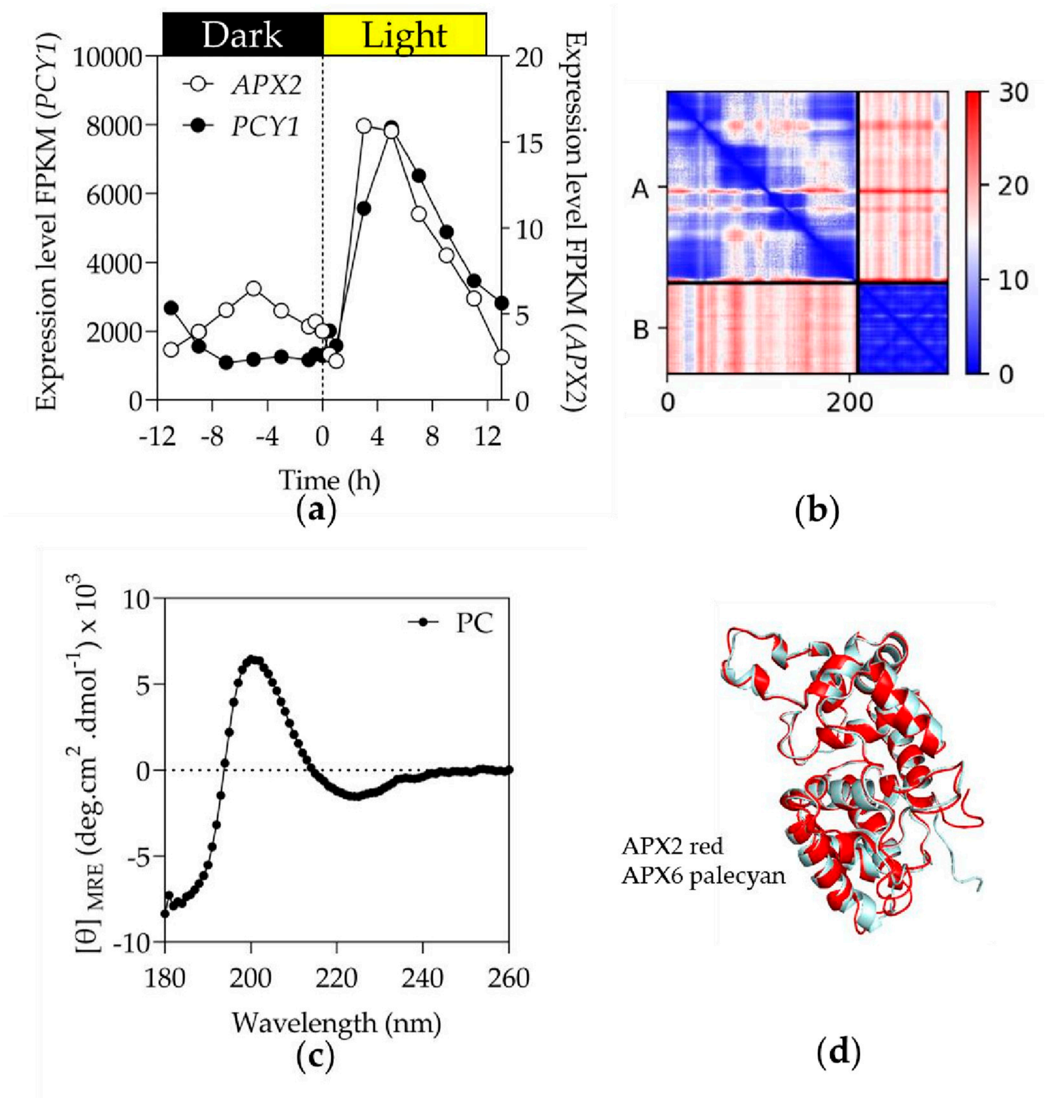

Figure S4. Further analyses of APX2 and plastocyanin. (**a**) Transcript levels of APX2 and plastocyanin (PCY1) in Chlamydomonas present similar dynamics changes in the light phase of asynchronized day-night culture. The time points -11 to 0 correspond to the dark phase, the time points 0 to 12 corresponds to the light phase, time points 12 to 13 correspond to the next dark phase. The data are retrieved from [4]. (**b**) AlphaFold2_multimer pairwise alignment diagram for the interaction of APX2 with apoplastocyanin. A represents all the residues of APX2 and B represents all the residues of apoplastocyanin. AF2 predicted five models and only the one ranked as number one is shown. The first quadrant shows the prediction error on the interactions within APX2 and the fourth quadrant the internal interaction error within apoplastocyanin. The blue color indicates high confidence, while the red color indicates low confidence (or high flexibility). The blue line marks in the second and third quadrant of the diagram show specific well predicted interactions between both proteins. (**c**) Circular dichroism spectrum of the recombinant plastocyanin. Circular dichroism between 180 and 260 nm of recombinant plastocyanin (PC, 50 μM) in 20 mM phosphate buffer, pH 7.4 + 100 mM NaF shows the characteristic folded structure (recombinant plastocyanin was a kind gift from M. Hippler and Yuval Milrad (UMunster)). (**d**) Superimposition of predicted structure of APX2 of Chlamydomonas (red) and APX6 of Arabidopsis (palecyan) (predicted with AlphaFold2 powered by ColabFold) reveals that the two proteins are similar. The r.m.s.d. is 0.407 Å.

References

1. Dunand, C.; Mathé, C.; Lazzarotto, F.; Margis, R.; Margis-Pinheiro, M. Ascorbate peroxidase-related (APx-R) is not a duplicable gene. *Plant Signal. Behav.* **2011**, *6*, 1908–1913, https://doi.org/10.4161/psb.6.12.18098.
2. Raven, E.L. Understanding functional diversity and substrate specificity in haem peroxidases: what can we learn from ascorbate peroxidase? *Nat. Prod. Rep.* **2003**, *20*, 367–381, https://doi.org/10.1039/b210426c.
3. Jumper, J.; Evans, R.; Pritzel, A.; Green, T.; Figurnov, M.; Ronneberger, O.; Tunyasuvunakool, K.; Bates, R.; Žídek, A.; Potapenko, A.; et al. Highly accurate protein structure prediction with AlphaFold. *Nature* **2021**, *596*, 583–589, https://doi.org/10.1038/s41586-021-03819-2.
4. Strenkert, D.; Schmollinger, S.; Gallaher, S.D.; Salomé, P.A.; Purvine, S.O.; Nicora, C.D.; Mettler-Altmann, T.; Soubeyrand, E.; Weber, A.P.M.; Lipton, M.S.; et al. Multiomics resolution of molecular events during a day in the life of Chlamydomonas. *Proc. Natl. Acad. Sci.* **2019**, *116*, 2374–2383, https://doi.org/10.1073/pnas.1815238116.
